# Tertiary lymphoid structures associated with enhanced anti-tumor immunity and favorable prognosis in cervical squamous carcinoma

**DOI:** 10.18632/aging.205733

**Published:** 2024-04-17

**Authors:** Guohai Xiong, Jinmei Shan, Qingguo Chong, Yueqing Cui

**Affiliations:** 1 Department of Gynaecology, Yancheng Third People’s Hospital, Yancheng 224008, China; 2 Department of Gynaecology, The Yancheng School of Clinical Medicine of Nanjing Medical University, Yancheng 224008, China

**Keywords:** tertiary lymphoid structures, anti-tumor immunity, cervical cancer, prognosis

## Abstract

Background: Cervical squamous carcinoma (CESC) is the main subtype of cervical cancer. Unfortunately, there are presently no effective treatment options for advanced and recurrent CESC. Tertiary lymphoid structures (TLSs) are clusters of lymphoid cells that resemble secondary lymphoid organs; nevertheless, there is no summary of the clinical importance of TLS in CESC.

Methods: A large set of transcriptomic and single-cell RNA-sequencing (scRNA-seq) datasets were used to analyze the pattern of TLS and its immuno-correlations in CESC. Additionally, an independent in-house cohort was collected to validate the correlation between TLS and TME features.

Results: In the current study, we found that the presence of TLS could predict better prognosis in CESC and was correlated with the activation of immunological signaling pathways and enrichment of immune cell subpopulations. In addition, TLS was associated with reduced proliferation activity in tumor cells, indicating the negative correlation between TLS and the degree of malignancy. Last but not least, in two independent immunotherapy cohorts, tumors with the presence of TLS were more sensitive to immunotherapy.

Conclusion: Overall, TLS is related to an inflamed TME and identified immune-hot tumors, which could be an indicator for the identification of immunological features in CESC.

## INTRODUCTION

As one of the most common gynecological cancers, cervical cancer has become a major public health concern and poses a serious risk to patients’ lives. According to statistics from the American Cancer Society, cervical cancer will cause 4,310 cancer-related deaths this year. In addition, incidence and mortality both rank third among all female cancers [1]. Given that the human papillomavirus is recognized as a prominent etiological factor, cervical squamous carcinoma (CESC), the most prevalent subtype of cervical cancer, is to some extent avoidable [2]. Effective therapeutic options for advanced and recurring CESC are still missing, despite the fact that comprehensive therapeutic schemes that combine surgery, radiation, chemotherapy, and targeted therapy have had considerable success [3]. Unfortunately, the prognosis for patients with advanced cancers is typically unfavorable. Therefore, it is imperative for both clinicians and patients that further research should be conducted on treatment monitoring and prognostic assessment of CESC.

Tertiary lymphoid structures (TLSs) are collections of lymphoid cells and similar to that of secondary lymphoid organs [4]. Both structures share similar developmental traits, despite the fact that TLSs typically grow in non-lymphoid tissues that are continuously inflamed, such as malignant tissues [5]. Immune cells of various sorts, representing various stages of development, are seen in TLSs with differing degrees of spatial organization, and these structures assist both humoral and cellular immune responses [6, 7]. The relationship between the presence of TLS and the prognosis in cancer patients has been thoroughly researched [8]. For example, B cells in the TLSs mediated the immunological status and promoted anti-tumor response by producing disease-relevant antibodies, which can mark antigen-expressing cells for opsonization, complement-mediated lysis, or antibody-dependent cellular cytotoxicity [9]. Also, T cells within TLSs had higher levels of activation markers [10], and mediated the recruitment of CD8+ T cells to improve the survival [11]. Many studies observed that the existence of TLS was related to better survival and well therapeutic responses in many solid carcinomas [12–16]. In addition, the discovery that TLSs are linked to favorable clinical outcomes in some cancer types has also prompted the development of treatment strategies [5]. However, the clinical significance of TLS in CESC has not been summarized.

Therefore, in this study, we described the clinical significance of TLS in CESC using multiple public and in-house cohorts. As results, we found that TLS was related to well prognosis and immuno-hot tumor microenvironment (TME). In addition, TLS was also associated with low proliferation index in CESC. Moreover, TLS could predict the immunotherapeutic responses in multiple cancer types, including breast cancer and melanoma. Overall, TLS is a biomarker to identify immuno-hot tumors and predict clinical outcome in CESC.

## METHODS

### Dataset gathering

The transcriptional omics and clinical annotations of CESC cohorts were obtained from The Cancer Genome Atlas (TCGA) and the Gene Expression Omnibus (GEO, ID: GSE44001 dataset [17]) The normalized gene expression profiles of clinical cohorts of patients with anti-PD-1 therapy from the GSE194040 [18] and the PRJEB23709 [19] cohorts were also obtained from the public databases. Samples with unclear survival information were removed from this study. Diagnostic patients with immunotherapeutic responses were selected for further analysis. In addition, the gene signature of TLS was obtained from Cabrita et al.’s study [20]. The information of datasets used in the study was listed in Supplementary Table 1.

### Single-cell RNA sequencing datasets analysis

The single-cell RNA sequencing (scRNA-seq) datasets of CESC patients from the GSE171894 and the GSE168652 [21] datasets were downloaded from the GEO database. The R package Seurat [22], were utilized for all additional analysis, such as quality control, unsupervised clustering and cell annotations. Given the influence of technical background noise, we removed abnormal cells in which the expression of mitochondrial genes was greater than 10% or with detected genes less than 200 or greater than 5,000. Subsequently, a total of 20,117 individual cells were from five CESC patients that passed the quality control. Then, the “RunHarmony” function in the R package harmony [23] was used to minimize the technical batch effects among individuals and experiments. To reduce the high dimensional datasets to two dimensions, the principal component analysis (PCA) was firstly performed on the top 4,000 genes with the highest variants, and then the first 30 PCs were used to reduce the dimensionality. The shared nearest neighbor modularity optimization-based clustering algorithm was used to unsupervised these cells into many clusters with a resolution of one. t-Distributed Stochastic Neighbor Embedding (t-SNE) was used to visualize the distribution of cells at the two-dimensional space.

### Identification of differential expressed genes (DEGs)

The R package “limma” was used to perform the differential expression analysis. Genes with the |FC| ≥ 1.5 and adjusted *P*-values < 0.05 were defined as the DEGs.

### Immunoinfiltration assessment

The ESTIMATE algorithm [24] was utilized to infer the relative abundance of tumor cells, stromal cells, and immune cells of tumor tissues. To assess the immune status of TME, many signatures of immunological features, including immunomodulators [25], the 29 immune cell types and immune-related pathways [26] were obtained from previous studies. The single-sample enrichment analysis (ssGSEA) algorithm was performed to estimate the enrichment levels of these features.

### Functional and pathway enrichment analyses

The R package clusterProfiler package [27] was performed to investigate the enriched biological pathways based on the up-regulated genes. Gene Ontology (GO) [28] and Kyoto Encyclopedia of Genes and Genomes (KEGG) [29] terms were identified with a strict cutoff of *p*-value < 0.05.

### Collection of clinical samples

Paraffin-embedded cervical cancer tissue microarray (TMA) (catalog HUteS168Su01) was obtained from the National Engineering Center for Biochip (Shanghai Outdo Biotech, Shanghai, China). Detailed clinic-pathological features and follow-up data were obtained from Outdo Biotech. Ethical approval for the use of TMAs was granted by the Clinical Research Ethics Committee at Outdo Biotech (SHYJS-CP-1710003). In addition to adenocarcinoma and adenosquamous carcinoma samples, a total of 110 CESC samples were included in our study.

### Cancer tissues staining and assessment

Immunohistochemistry (IHC) staining and Hematoxylin and Eosin (HE) staining were performed on the above TMAs and tissue slides. For IHC and HE staining, the standard operating procedures were used. The primary antibodies applied in the research were as follows: anti-PD-L1 (ready-to-use, catalog GT2280, GeneTech, Shanghai, China) and anti-KI67 (ready-to-use, catalog GT2101, GeneTech, Shanghai, China). Antibody staining was visualized with diaminobenzidine and hematoxylin counterstain. TLS was blindly quantified by two senior pathologists without the knowledge of clinic-pathological features. The presence and location of TLS were assessed based on morphology in HE staining sections. For quantitative evaluation of PD-L1 and KI67 staining, two senior pathologists scored PD-L1 and KI67 expression in tumor cells based on the immunoreactivity score (IRS) criterion [30].

### Statistical analysis

R software (version 4.1.2) was utilized for all statistical analyses. Wilcoxon rank sum test was used for the difference between two groups of continuous variables, and Fisher exact test was used for the difference between categorical variables. For all analyses, a two-paired *p*-value ≤ 0.05 was deemed to be statistically significant, and labeled with ^*^p-value ≤ 0.05, ^**^p-value ≤ 0.01, ^***^p-value ≤ 0.001, and ^****^p-value ≤ 0.0001.

### Data availability statement

The TCGA data are openly available at https://portal.gdc.cancer.gov/. The GEO data are openly available at https://www.ncbi.nlm.nih.gov/gds.

## RESULTS

### Expression pattern of TLS-related genes in CESC

To describe the expression patterns of TLS-related genes in CESC, the gene lists were obtained from Cabrita et al.’s study [20]. Then, a total of 9 TLS-related genes (EIF1AY, CETP, PTGDS, RBP5, CD1D, SKAP1, CD79B, LAT, and CCR6) were used to estimate the TLS score using the GVAS algorithm (Supplementary Figure 1A). The mutation rate of these 9 genes was low, indicating these genes were stable (Supplementary Figure 1B). We also checked the copy number variation (CNV) of these 9 genes and found that CD1D exhibited the highest amplification ratio (more than 50%) and CCR6 exhibited the lowest deletion ratio (nearly 40%) (Supplementary Figure 1C).

**Figure 1:**
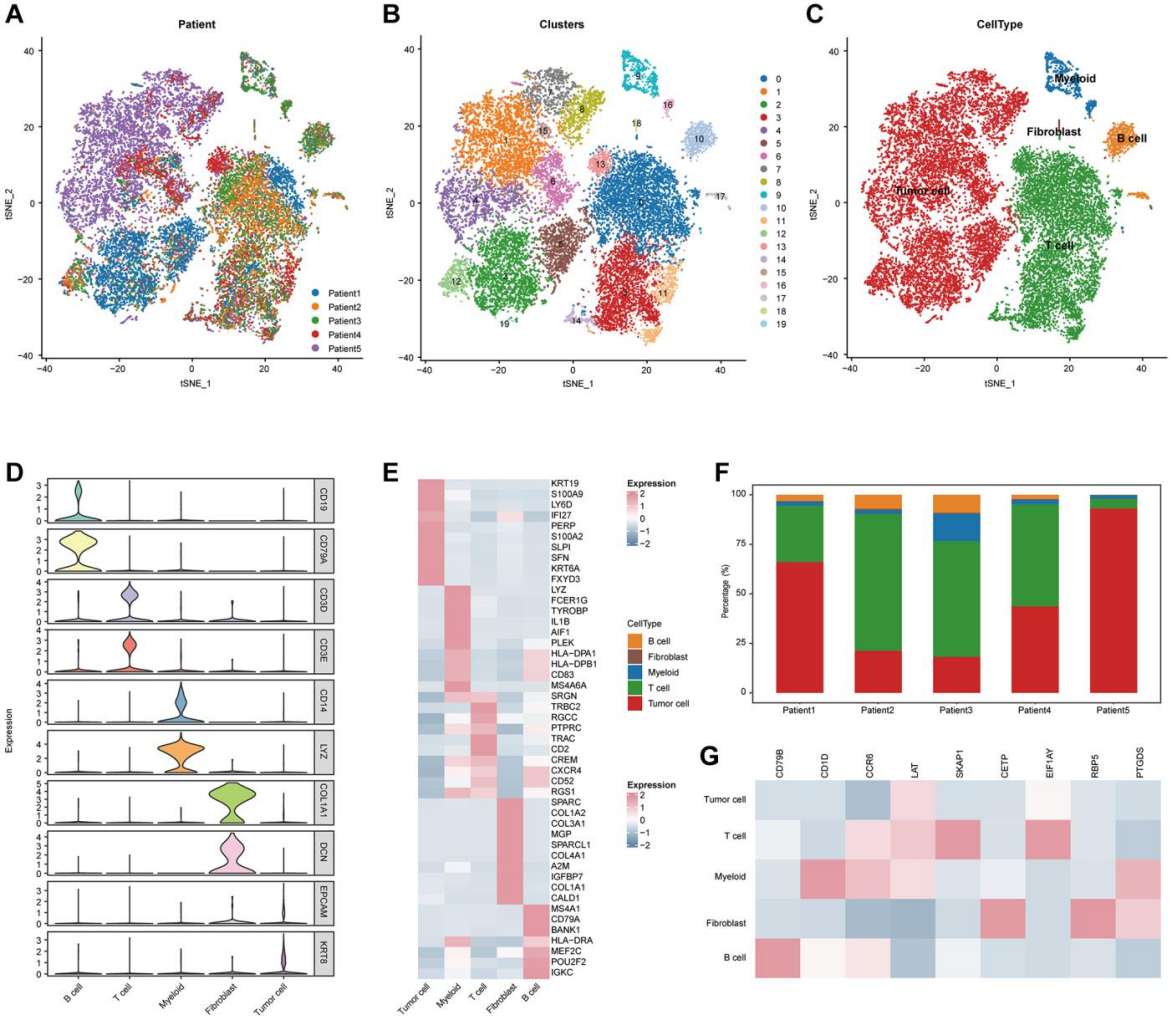
**Integrated scRNA-seq analysis of tumor tissues from CESC patients.** (**A**) t-SNE visualization of 20,117 single cells passed quality controls, colored by three CESC patients. (**B**) Unsupervised clustering of 20,117 cells. (**C**) t-SNE visualization of major cell types. (**D**) Violin plot showing the expression levels of conventional gene signatures. (**E**) Heatmap for gene expression levels of top ten cell-type-specific genes. (**F**) Bar plot showing the cellular components of each CESC patients. (**G**) Heatmap showing the expression of nine TLS signatures of the five major cell types.

Next, to characterize TLS-related genes in CESC at the single-cell level, we collected two scRNA-seq datasets (the GSE171894 and the GSE168652 datasets) (Figure 1A). As results, individual cells were passed quality control criteria and were unsupervised clustered into 19 clusters (Figure 1B). These clusters were explored with unbiased clustering across all cells by PCA and visualized by the t-SNE analysis. We annotated the cell type of each cluster with the conventional gene markers, including tumor cells, B cells, T cells, fibroblasts, and myeloid cells (Figure 1C). To further support the single cell annotation, the genes that are exclusively expressed in each type of cell were found (Figure 1D–1F, Supplementary Figure 2). Next, we estimated the expression of 9 TLS-related genes in individual cells, and the results showed that all these genes were highly expressed in various immune cells, such as CD79B was highly expressed in B cells and CD1D was highly expressed in myeloid cells (Figure 1G). All findings confirm that TLS-related genes are highly expressed in immune cells.

### TLS was associated with well prognosis and inflamed TME in CESC

**Figure 2:**
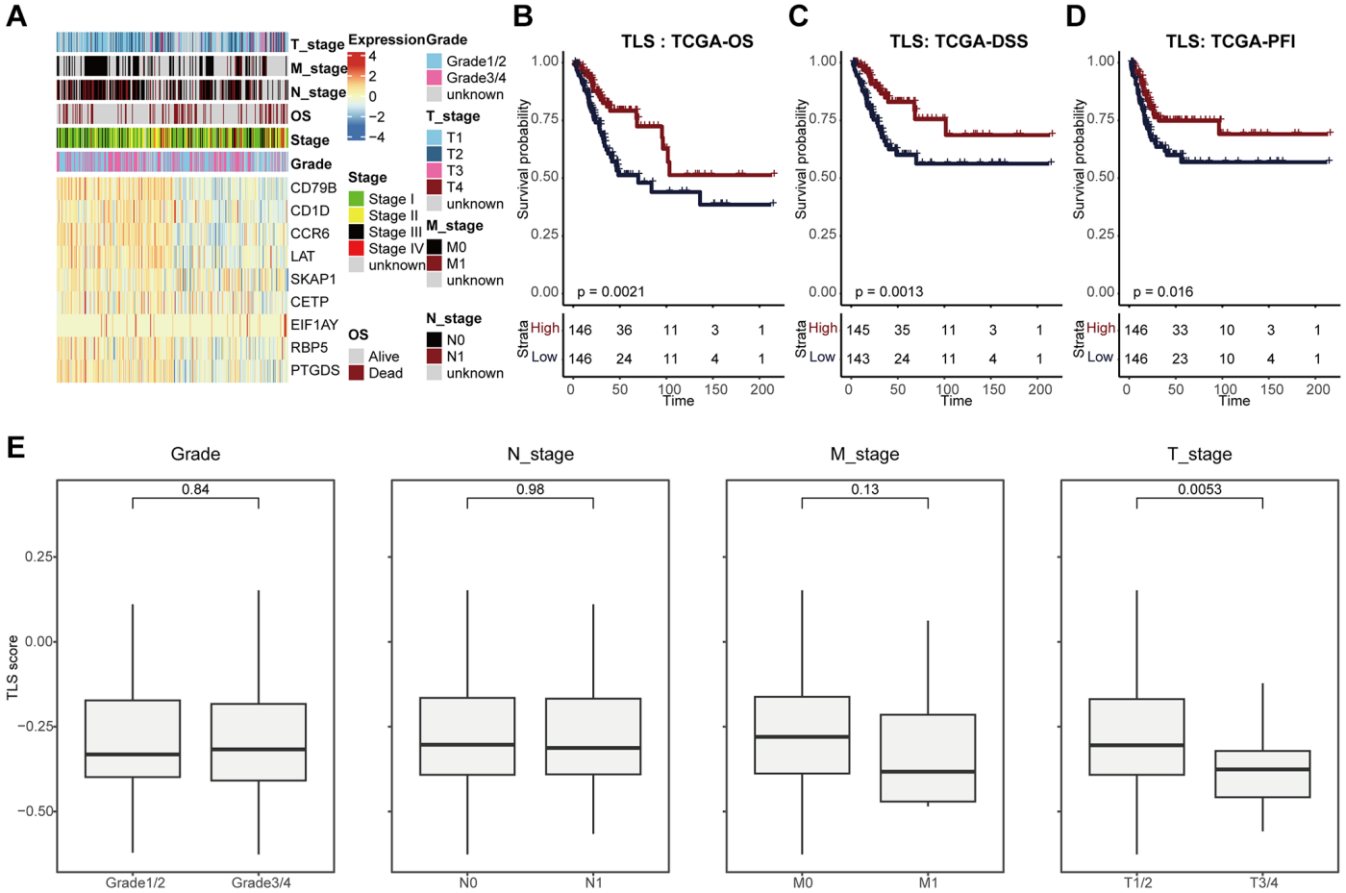
**TLS was associated with favorable clinical outcome of CESC.** (**A**) Heatmap showing the expression of nine TLS signatures in the TCGA-CESC cohort. (**B**–**D**) Kaplan-Meier curves showing the (**B**) OS-related, (**C**) DSS-related, (**D**) PFI-related predictive values of TLS in the TCGA-CESC cohort. Patients were divided into two groups based on the median value of TLS. (**E**) Comparing TLS among patients with different clinic-pathological characteristics.

Next, we analyzed the associations between TLS and clinical features (Figure 2A, 2E). We found that TLS score was higher in tumors with earlier T stage, but not related to other features, such as N stage, M stage, and differentiation degree (Figure 2E). In addition, high TLS score was significantly associated with well prognosis in terms of OS, PFS, and DSS (Figure 2B–2D), and the predictive value of TLS for prognosis was higher than single TLS-related gene (Supplementary Figure 3A–3C).

**Figure 3:**
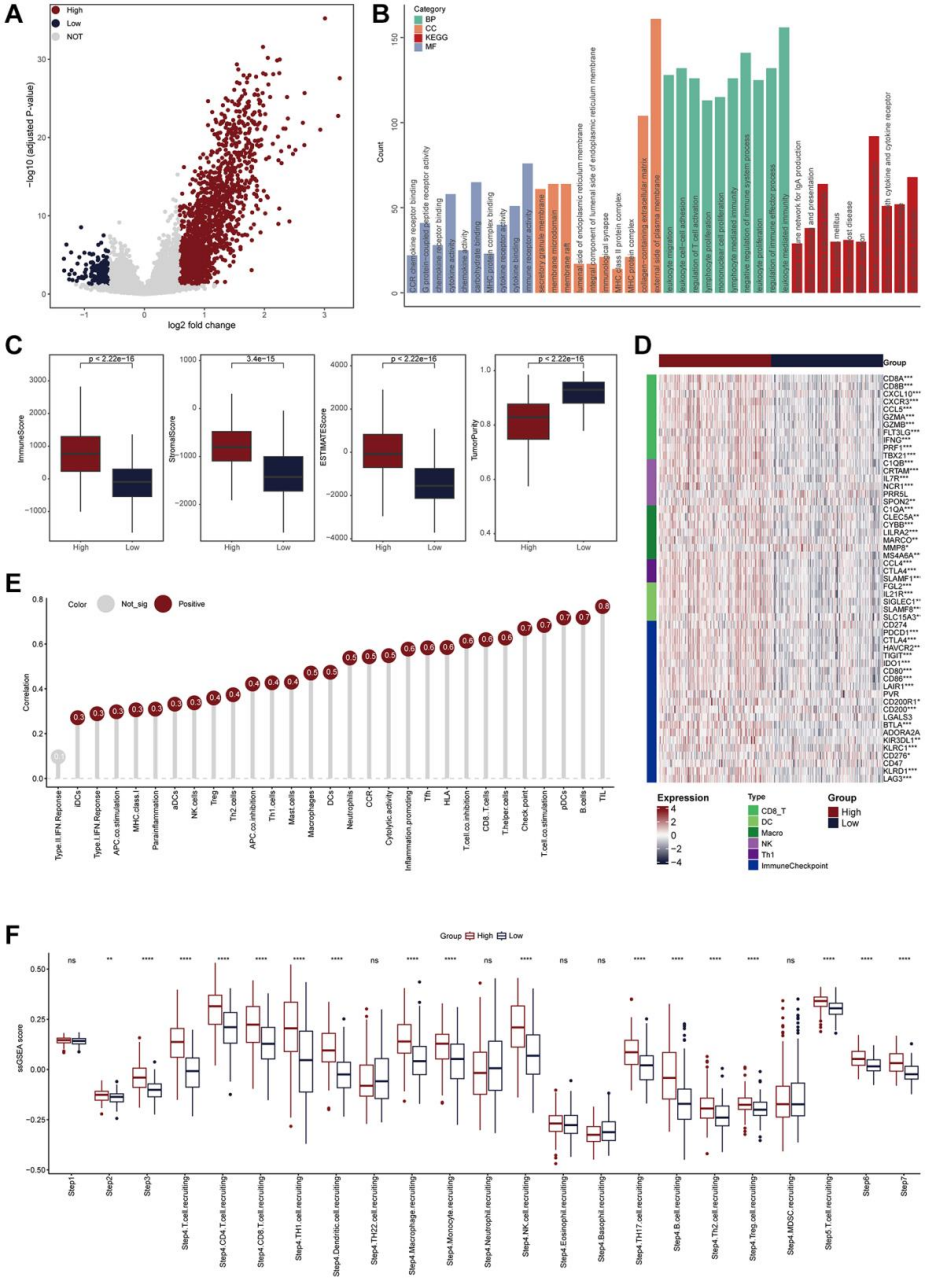
**TLS was positively correlated with immunological scores in the TCGA-CESC cohort.** (**A**) Volcano plot showing the DEGs for the TLS-high and low groups in the TCGA-CESC cohort. Red point: the up-regulated genes of TLS-high group. Blue point: the up-regulated genes of TLS-low group. Grey point: genes with no statistical significance. (**B**) Biological pathways enriched in the TLS-high group. (**C**) Comparison of StromalScore, ImmuneScore, ESTIMATEScore and tumor purity between TLS-high and low groups. (**D**) Heatmap showing the expression of biomarkers of immune subpopulations and immune checkpoints in the TCGA-CESC cohort. (**E**) The correlation between TLS expression and the enrichment scores of immune subpopulations and immune-related signaling pathways in the TCGA-UCEC cohort. (**F**) Comparing the enrichment scores of each step in the cancer immunity cycle between TLS-high and low groups.

**Figure 4:**
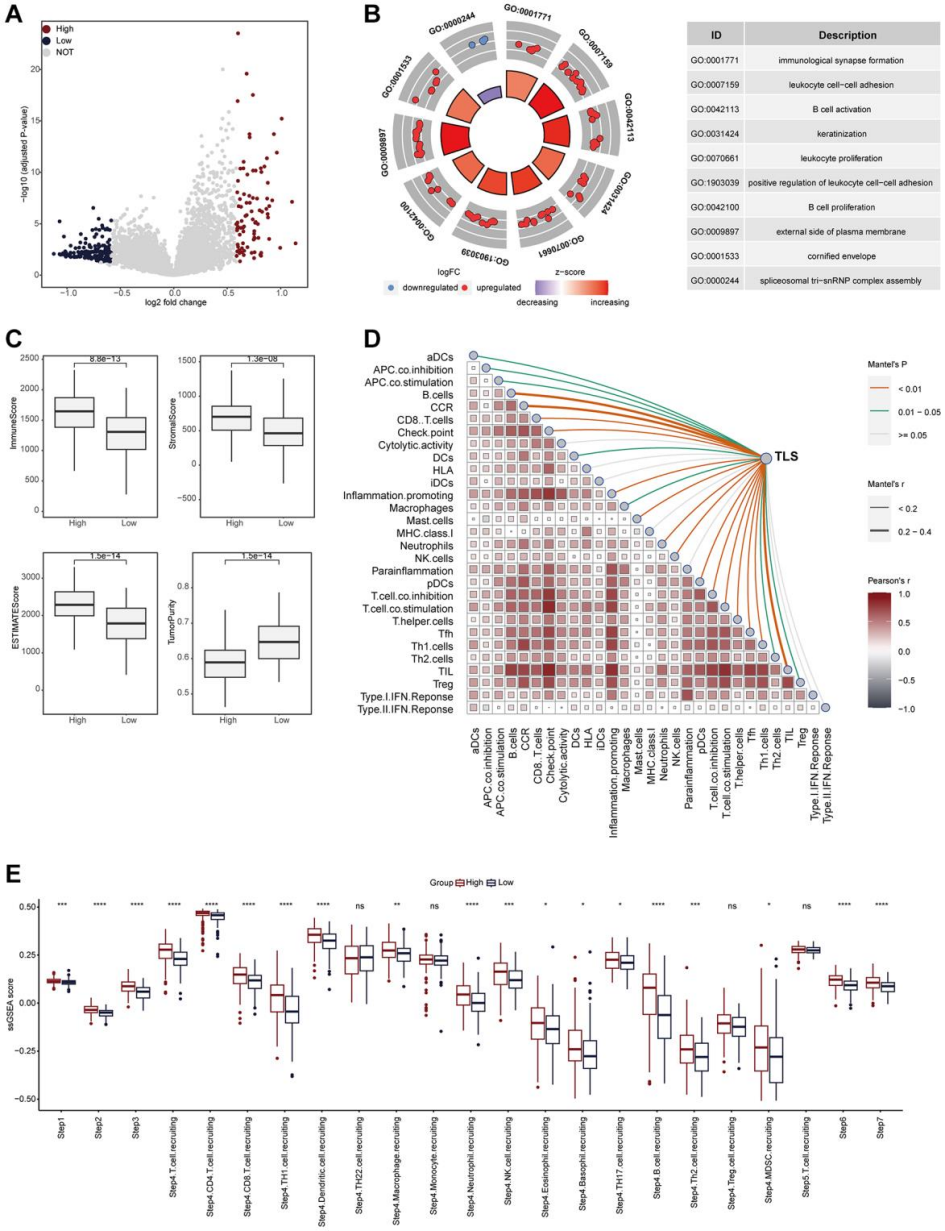
**TLS was positively correlated with immunological scores in the GSE44001 cohort.** (**A**) Volcano plot showing the DEGs for the TLS-high and low groups in the TCGA-CESC cohort. (**B**) Biological pathways enriched in the TLS-high and low groups, respectively. Red dots represented the genes up-regulated in TLS-high group. Blue dots represented the genes down-regulated in TLS-low group. (**C**) Comparison of StromalScore, ImmuneScore, ESTIMATEScore and tumor purity between TLS-high and low groups. (**D**) The correlation between TLS and the enrichment scores of immune subpopulations and immune-related signaling pathways in the TCGA-UCEC cohort. (**E**) Comparing the enrichment scores of each step in the cancer immunity cycle between TLS-high and low groups.

We also explored the TLS-related molecular mechanisms in CESC. DEGs between low- and high-TLS score were screened (Figure 3A). The results showed that highly expressed genes in the high-TLS score group were mainly enriched in immunity-related pathways (Figure 3B), and highly expressed genes in the low-TLS score group were mainly enriched in several oncogenic pathways, such as HIF-1 signaling pathway and PI3K-Akt signaling pathway (Supplementary Figure 4). In addition, TLS was positively correlated with ESTIMATE score and immune score, but negatively related to tumor purity (Figure 3C). By performing four independent algorithms, patients with TAP1-high phenotype had higher abundance of many immune cell types, such as CD8+ T cells, B cells and macrophages (Supplementary Figure 5). In view of the above results that TLS was correlation with anti-tumor immunity in CESC, we next investigated the exact correlation between TLS score and the landscape of tumor immunology. In the TCGA-CESC cohort, TLS was positively related to immuno-pathways and tumor-infiltrating lymphocytes (Figure 3D, 3E). Moreover, the positive correlation between TLS score and immunocirculatory activities was also observed in the TCGA-CESC cohort (Figure 3F). More importantly, the analysis of the GSE44001 cohort also confirmed the positive correlations between TLS score and activated anti-tumor immunity (Figure 4A–4E, Supplementary Figure 6A, 6B and Supplementary Figure 7A–7D).

**Figure 5:**
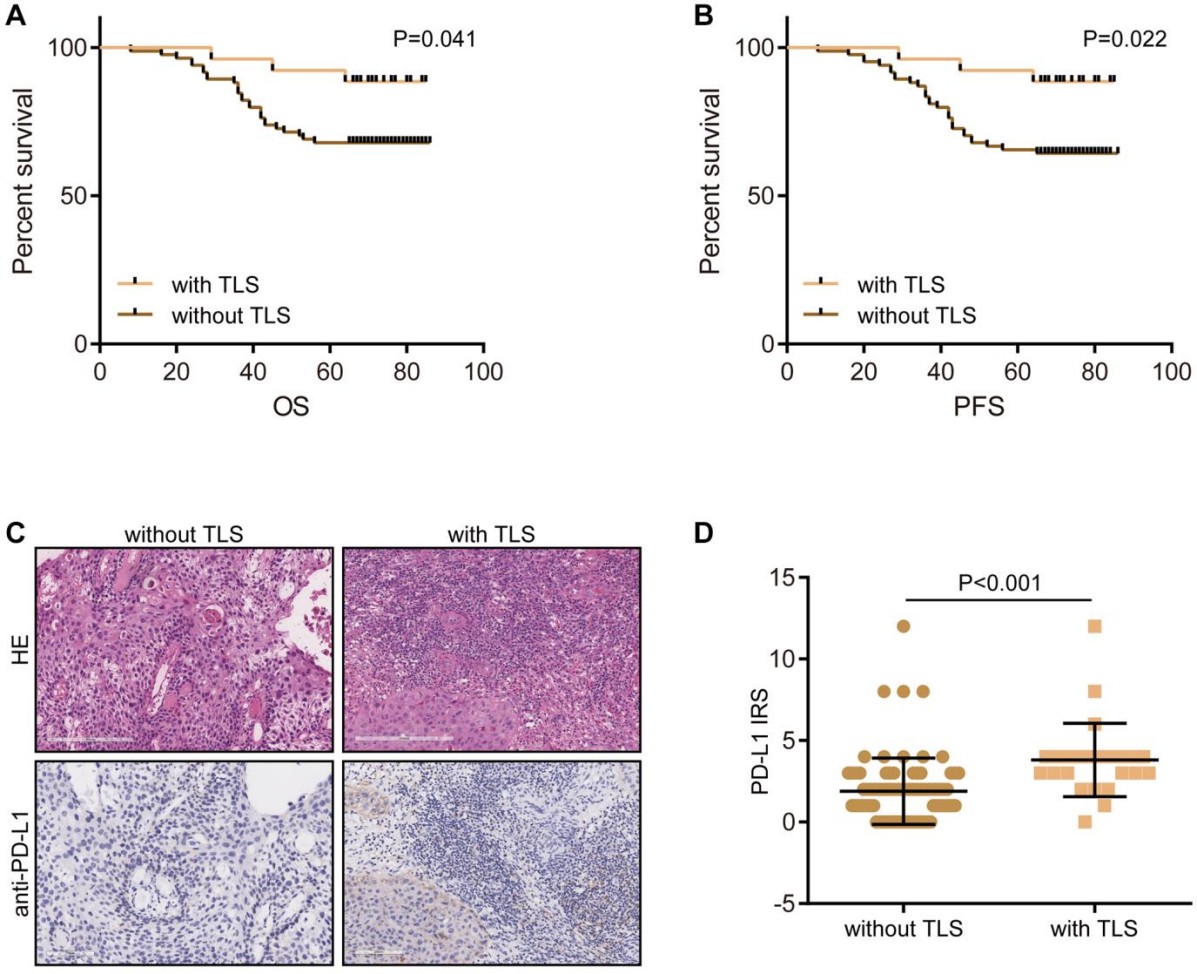
**Validation of the prognostic value of TLS and its association with TME features.** (**A**) The prognostic value of TLS in CESC in the term of OS. (**B**) The prognostic value of TLS in CESC in the term of PFS. (**C**, **D**) Representative images revealing PD-L1 expression in CESC samples with or without TLS and semi-quantitative analysis.

Moreover, to further verify the above findings, we recruited a CESC cohort consisting of a total of 110 CESC samples. In the in-house cohort, we found that the presence of TLS was significantly associated with well prognosis in terms of OS and PFS (Figure 5A, 5B). In addition, PD-L1, an immunotherapy biomarker, was highly expressed in samples with the presence of TLS (Figure 5C, 5D). Totally, all results suggest that TLS is associated with the shaping of an inflamed TME.

### TLS was associated with reduced proliferation activity in tumor cells

**Figure 6:**
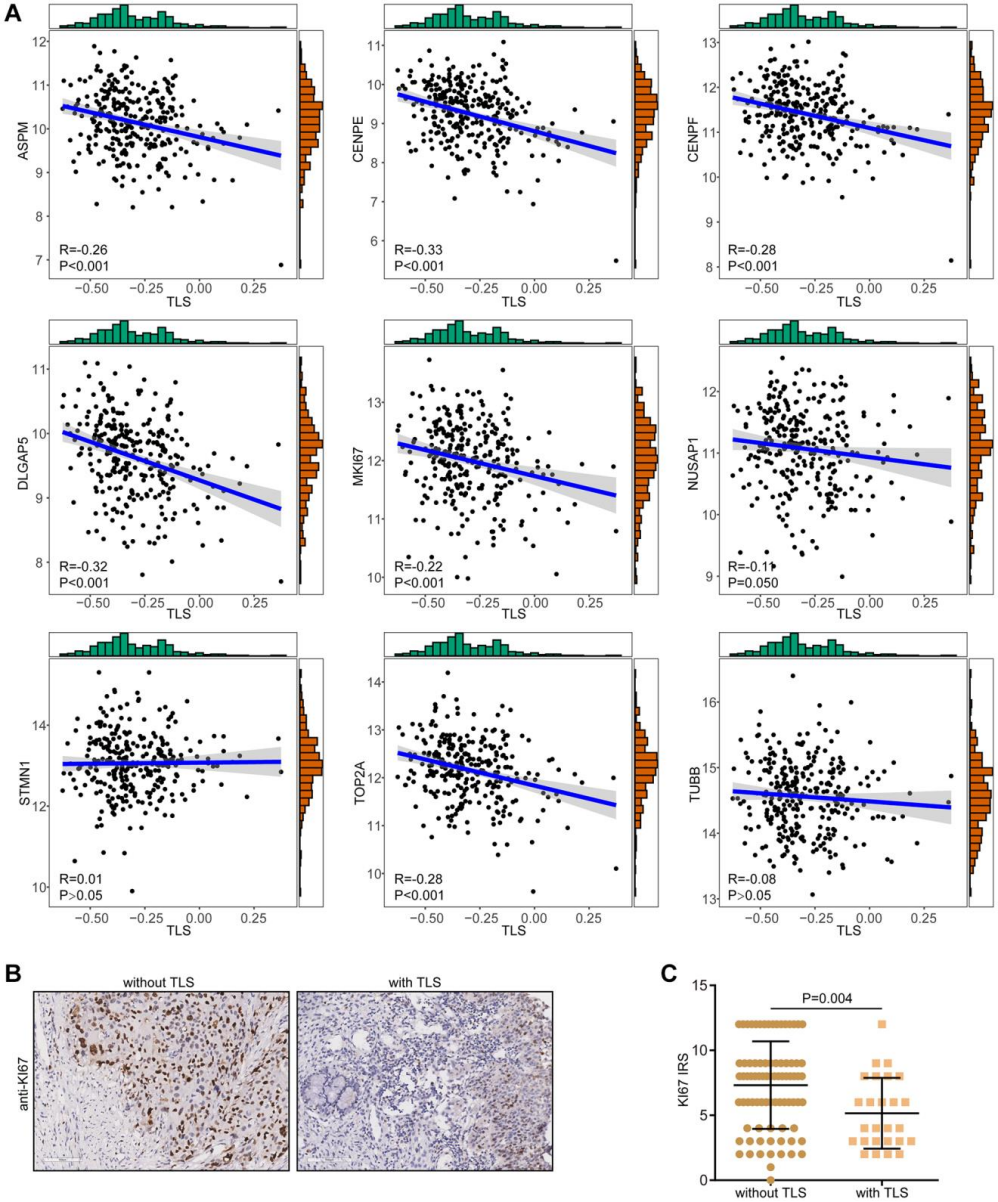
**Correlations between TLS and proliferation biomarkers.** (**A**) Correlations between TLS score and a total of nine proliferation biomarkers in the TCGA-CESC cohort. (**B**, **C**) Representative images revealing KI67 expression in CESC samples with or without TLS and semi-quantitative analysis.

Given the potential correlation between TLS and oncogenic pathway (Supplementary Figure 4, Supplementary Figure 6B), we speculated that TLS might be associated with malignancy of tumor cells. Proliferation activity is the most important feature tumor cells. The results showed that TLS score was negatively correlated with proliferation indexes in both the TCGA-CESC and the GSE44001 cohorts (Figure 6A, Supplementary Figure 8). In addition, we also validated the negative correlation between TLS and proliferation activity (Figure 6B, 6C). In the in-house cohort, we found that tumors with the presence of TLS expressed low KI67 compared with these without TLS. Overall, these results may explain the correlation of the presence of TLS with better prognosis in CESC in the term of being independent of the TME.

### Tumors with the presence of TLS were sensitive to immunotherapy

**Figure 7:**
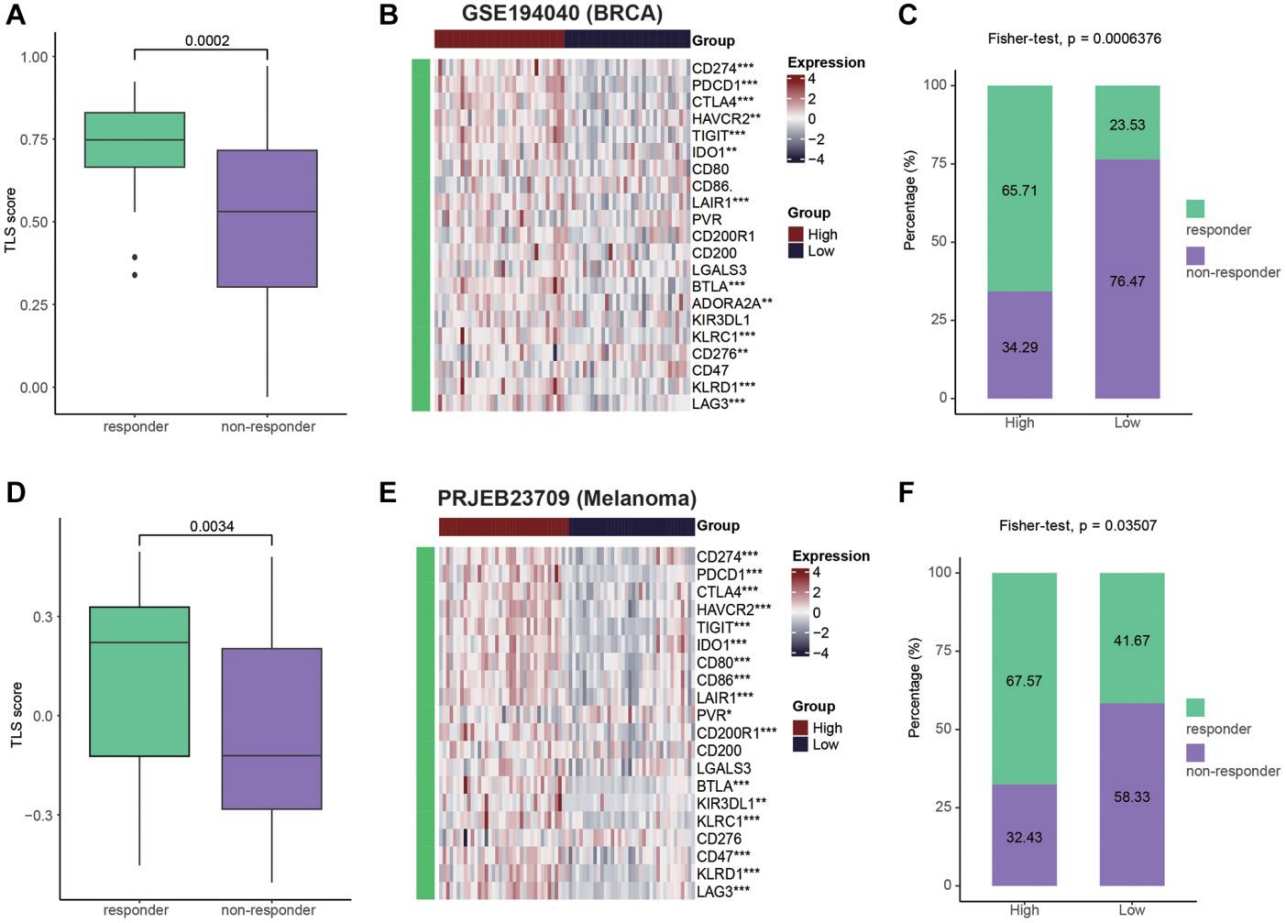
**Patients with high TLS were sensitive to immunotherapy.** (**A**) Comparing the TLS between responders and non-responders in the GSE194040 cohort (BRCA). (**B**) Heatmap showing the immune checkpoints expression between TLS-high and low groups in the GSE194040 cohort. (**C**) Bar plot showing the fractions of CR and NR patients in the TLS-high and low groups in the GSE194040 cohort. (**D**) Comparing the TLS between responders and non-responders in the PRJEB23709 cohort (Melanoma). (**E**) Heatmap showing the immune checkpoints expression between TLS-high and low groups in the PRJEB23709 cohort. (**F**) Bar plot showing the fractions of CR and NR patients in the TLS-high and low groups in the PRJEB23709 cohort. Wilcoxon rank-sum test was performed to measure the difference between two groups.

Given of the relationship between TLS and activation of immunological features, we wondered to know whether TLS could predict the immunotherapeutic responses. Due to the lack of sequencing data for gynecologic tumors for immunotherapy, we tested the predictive value of TLS for immunotherapy in breast cancer and melanoma. For breast cancer patients, compared with non-responders, tumors from responders had higher levels of TLS score (Figure 7A). In addition, high TLS score was related to high expression of immune checkpoints (Figure 7B). Encouragingly, the tumors from responders were enriched in the TLS-high group, while the tumors from non-responders tended to exhibit the TLS-low phenotype (Figure 7C). In melanoma, similar to breast cancer, tumors from responders had higher levels of TLS score, and TLS score was positively with immune checkpoints expression (Figure 7D, 7E). In addition, patients in the TLS-high group also showed better immunotherapeutic response than those in the TLS-low group (Figure 7F). Despite the association between TLS and favorable therapeutic response, for patients without TLS, there is still a lack of effective treatment options. Therefore, we collected the therapy-related signaling pathways from previous studies. Results showed that TLS-high group had higher levels of EGFR ligands and hypoxia-related pathways (Supplementary Figure 9A, 9B). All results suggest that patients with the presence of TLS tend to be sensitive to immunotherapy, while patients without TLS can benefit from anti-EGFR therapy and radiotherapy.

## DISCUSSION

In addition to tumor cells, solid tumors contain a significant amount of stromal cells, such as immune cells, fibroblasts, etc. [31]. According to the TME’s features, tumors might theoretically be classified as “hot” or “cold”. “Hot” tumors have higher response rates to these treatments and are characterized by T cell infiltration, increased interferon gamma signaling, high expression of inhibitory checkpoints, genomic instability, and the activation of major histocompatibility complex molecules. “Cold” tumors have an immunosuppressive TME and are resistant to either immunotherapy or chemotherapy [32–34]. Given that immune cell abundance is one of most significant factors that distinguish “hot” and “cold” tumors, the detection of immune cell characteristics is important for identifying “hot” and “cold” TME.

Immune cells, primarily B and T lymphocytes, form TLS in response to immunological stimuli. Different observations on the anti- and pro-tumor effects of TLSs in various cancer types reflect the various functions of TLSs in the initiation and development of tumors [35]. Recently, researchers have started to note the notable clinical significance of TLS in the TME. The prognostic value of TLS has been validated in numerous investigations on neoplasms [20, 36]. Previous research reported that the existence of TLS was related to better survival and well therapeutic responses in ovarian cancer [37, 38], head neck squamous cell carcinoma [12], breast cancer [13], oesophageal squamous cell carcinoma [14], and non-small cell lung cancer [39]. In CESC, Zhang et al. investigated the associations between TLS and immune features and found that TLS was strongly correlated with high level of PD-1 expression but there was no significant relationship between IL-33 and TLS [40]. However, the clinical significance and immuno-correlations of TLS in CESC has not been fully revealed.

In the current study, based on multiple public and in-house cohorts, we comprehensively described the clinical and immune correlations of TLS in CESC. Similar to findings in other tumor types, the existence of TLS was related to better prognosis in CESC. Remarkably, TLS was positively correlated with immuno-hot features, including low tumor purity, high ESTIMATEScore, ImmuneScore, and StromalScore, and high activities in cancer immunity cycle. Consistent with previous studies [11, 41], TLS scores were most positively correlated with the abundance of T and B cells at the bulk omics and single-cell transcriptional datasets. Some biological signaling pathways mediated by TLS, such as cytokine and chemokine activities, antigen processing and presentation [11, 20], were specifically activated in patients with high-TLS phenotype. Combined with these findings, our results indicated that T and B cells, the major component of TLS, may promote anti-tumor response and improve the survival by secreting cytokines and mediating antigen processing and presentation. More interestingly, we also found that TLS score was negatively correlated with tumor proliferation biomarkers, such as ASPM and KI67, which explained the correlation of the presence of TLS with better prognosis. Based on the data revealed by our research and previous study, the presence of TLS indicated the strong anti-tumor response, thus the proliferation of tumor cells could be inhibited [42]. However, in breast cancer, TLS presence was positively related to KI67 [43]. Whatever, this contradictory result still needs further investigation.

In addition to predicting prognosis of patients with cancers, TLS has been also reported to be related to immunotherapeutic responses in various cancer types, including cholangiocarcinoma [44], head neck squamous cell carcinoma [12], and non-small cell lung cancer [45]. In our study, we also found that TLS was associated with enhanced PD-L1 expression in CESC, a biomarker for immunotherapy [46]. However, due to the limited application of immunotherapy in CESC, we were unable to obtain the immunotherapy samples. Thus, we used public cohorts to assess the predictive value of TLS in breast cancer and melanoma, and found that high TLS score was associated with well immunotherapeutic responses.

## CONCLUSION

Overall, this research mainly focused on the clinical significance of TLS in CESC and revealed that TLS was tightly related to the well prognosis and activated anti-tumor immunity, which made it a promising indicator to the prognostic assessment and therapeutic guidance of CESC. The current study serves as a foundation for future research into the biology of TLS in CESC and lends support to the investigation of potential novel treatment avenues.

## Supplementary Materials

Supplementary Figures

Supplementary Table 1
