# Supplementary material for: Tertiary lymphoid structures associated with enhanced anti-tumor immunity and favorable prognosis in cervical squamous carcinoma
Source: Aging (Albany NY). 2024 Apr 17;16(8):6898–920. doi: 10.18632/aging.205733 (PMC11087108; doi:10.18632/aging.205733)
Supplement: Supplementary Figures [file aging-16-205733-s001.pdf]

SUPPLEMENTARY FIGURES

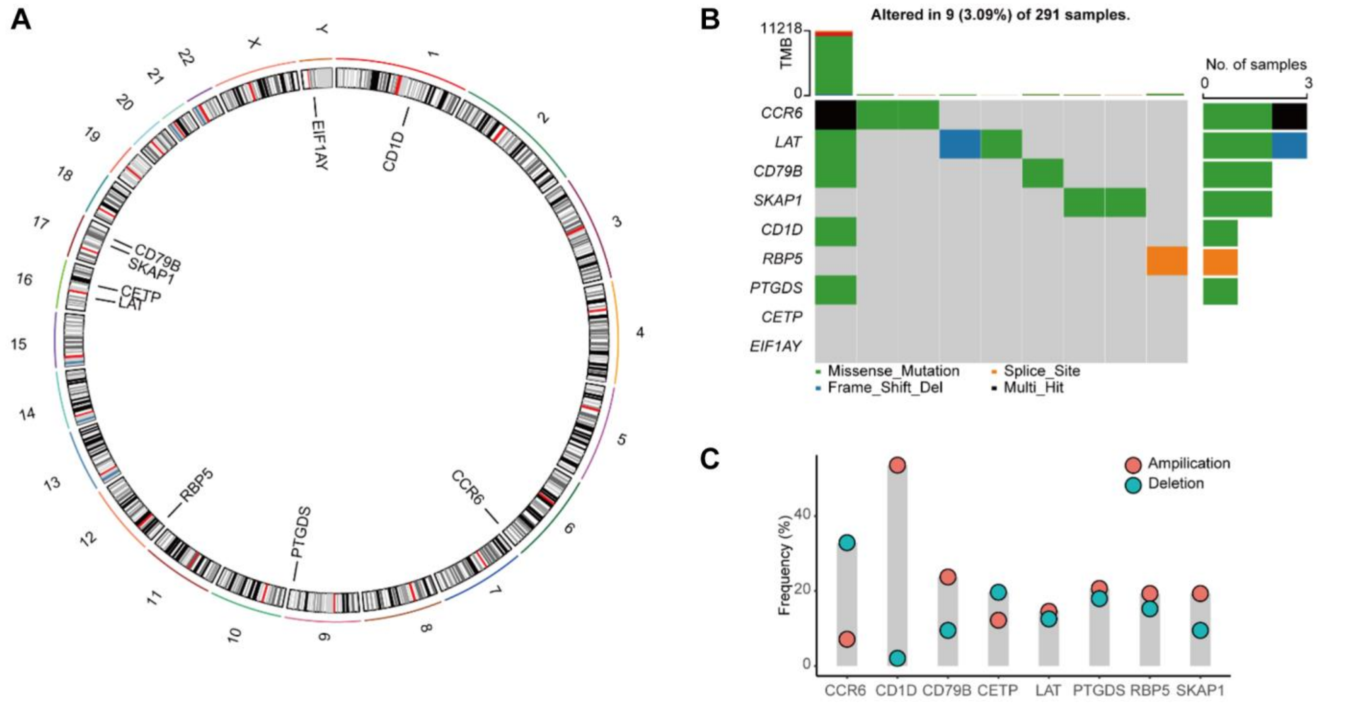

Supplementary Figure 1. Landscape of genetic variants of TLS signature genes in the TCGA-CESC cohort. (**A**) Location of nine TLS signature genes on 23 chromosomes. (**B**) Mutation frequency of nine TLS signature genes in the TCGA-CESC cohort. Each column represented a single patient. The upper bar plot showed TMB. The number on the right indicates the mutation frequency of each regulatory gene. The right bar plot showed the proportion of each variant type. The stacked barplot below showed a fraction of conversions in each sample. (**C**) CNV frequency of nine TLS signature genes in the TCGA-CESC cohort. The height of the column represented the alteration frequency. Blue dot: the deletion frequency; Red dot: the amplification frequency.

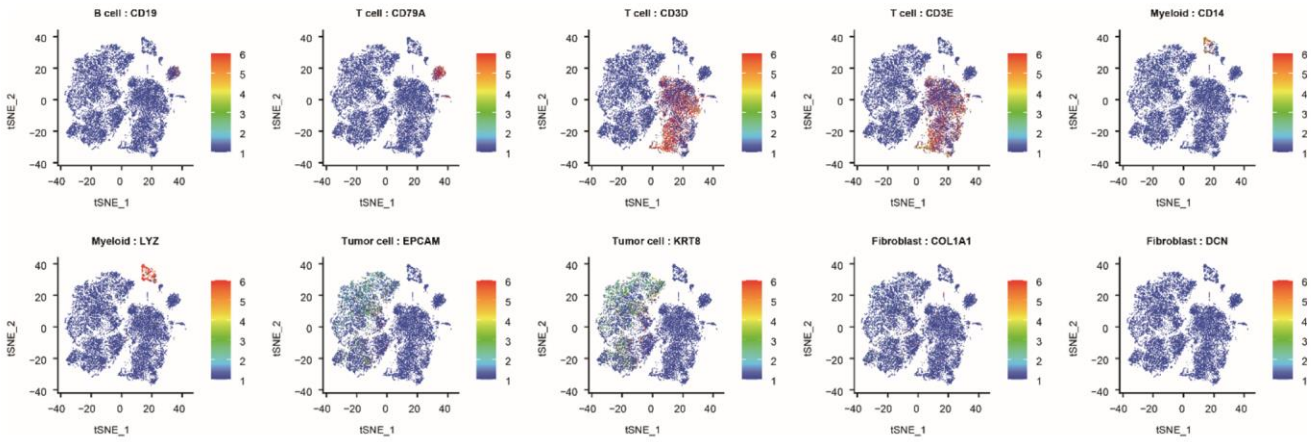

Supplementary Figure 2. Expression levels of known markers for specific cell types overlaid on the t-SNE representation.

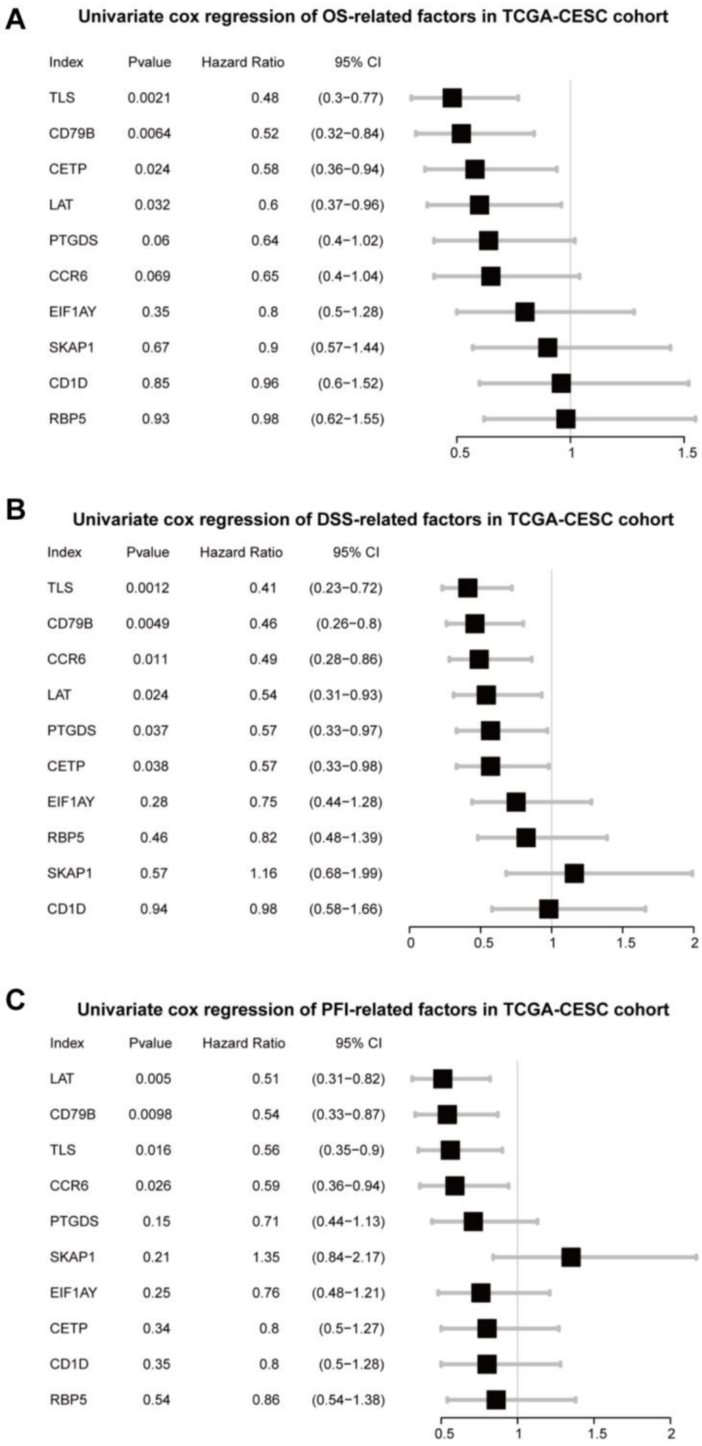

Supplementary Figure 3. Univariate Cox regression of TLS signatures in the TCGA-CESC cohort. Univariate Cox regression of (**A**) OS-related, (**B**) DSS-related, and (**C**) PFI-related TLS signatures in the TCGA-CESC cohort.

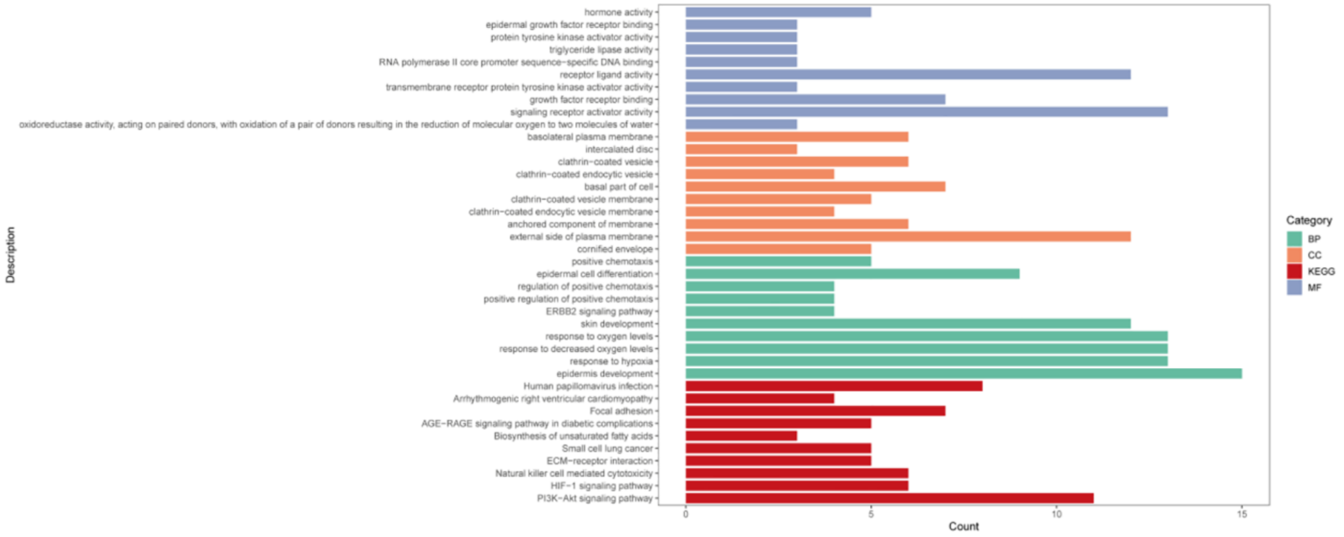

Supplementary Figure 4. Biological pathways up-regulated in the TLS-low group in the TCGA-CESC cohort.

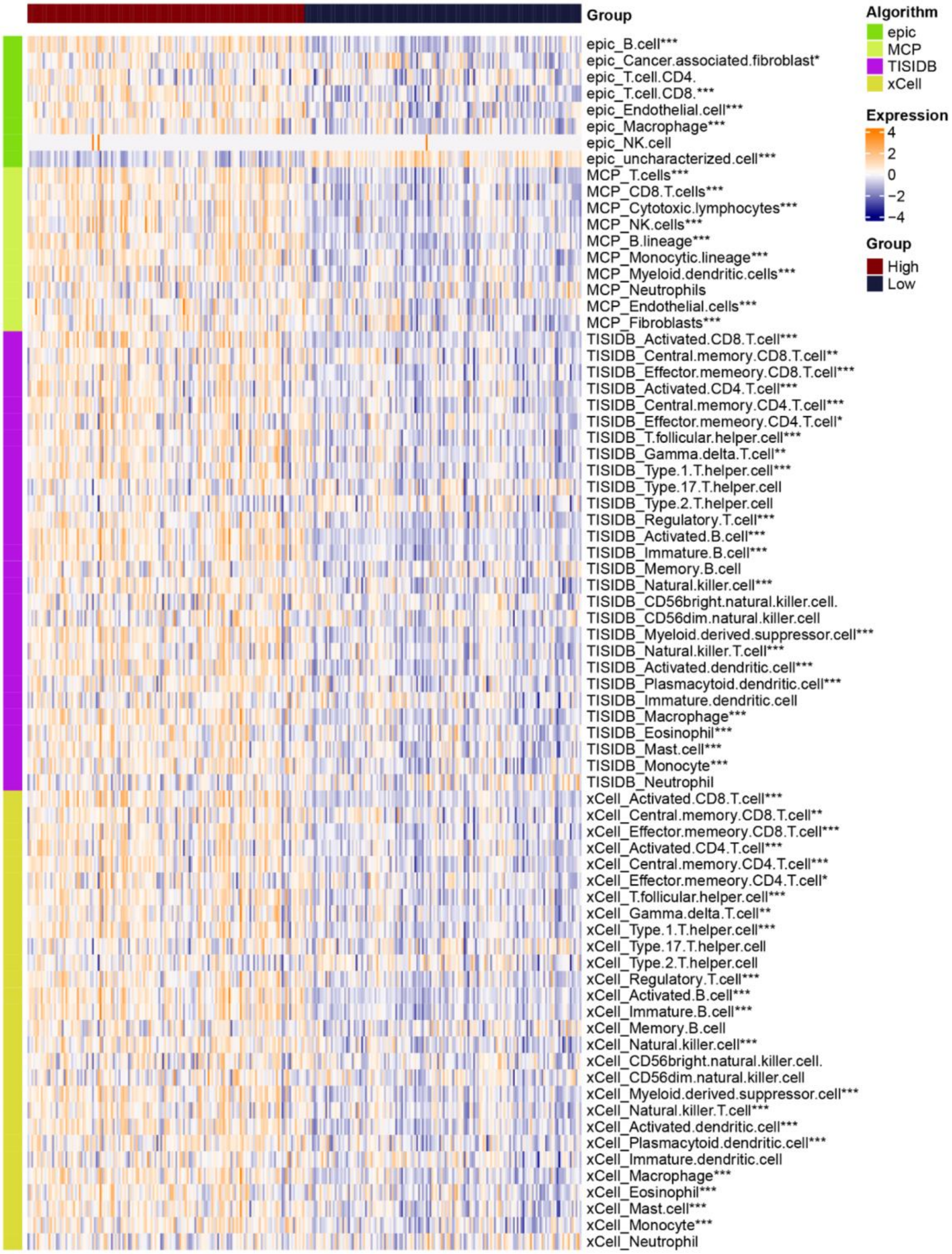

Supplementary Figure 5. Heatmap showing the relative abundance of cell types estimated by four independent algorithms in the TCGA-CESC cohort.

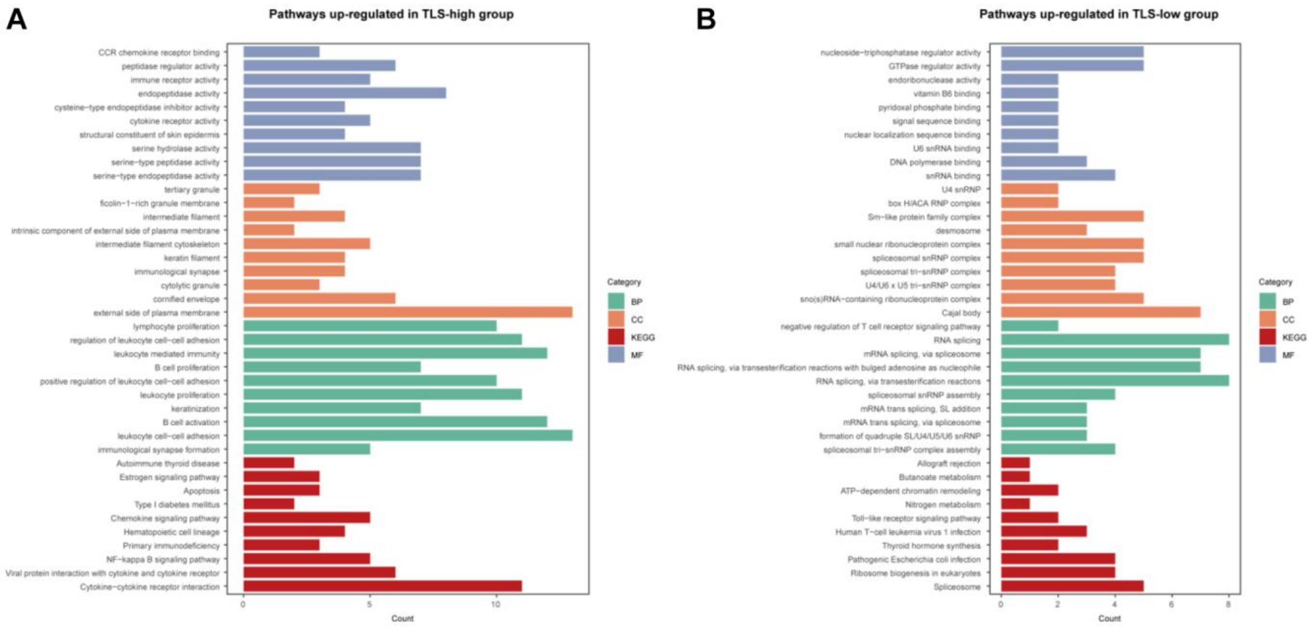

Supplementary Figure 6. Functional enrichment analyses in the GSE44001 cohort. (**A**) Biological pathways enriched in the TLS-high group. (**B**) Biological pathways enriched in the TLS-low group.

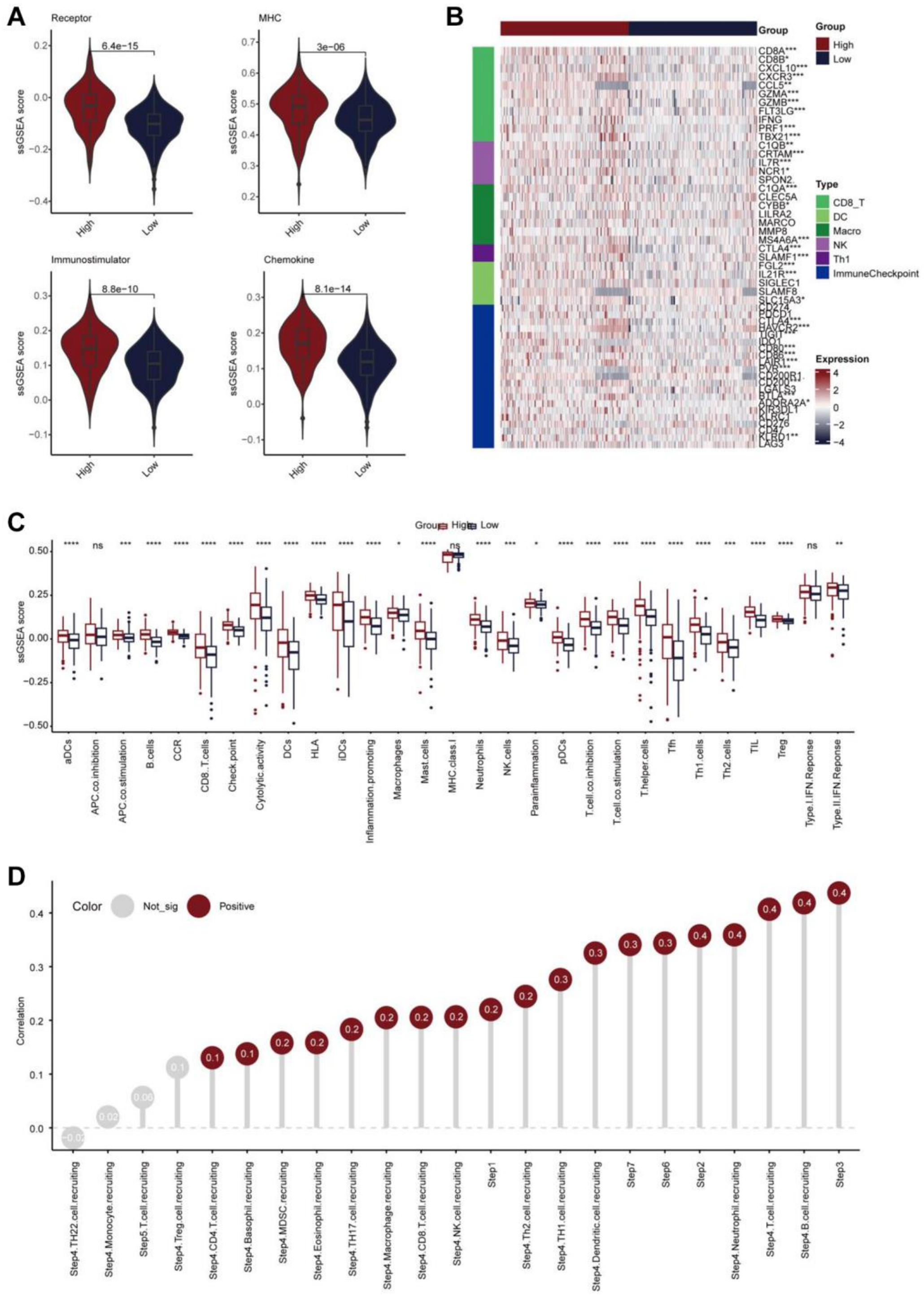

Supplementary Figure 7. Comparison of immunological characteristics between TLS-high and -low groups in the GSE44001 cohort. (**A**) Comparing StromalScore, ImmuneScore, ESTIMATEScore and tumor purity between TLS-high and -low groups. (**B**) Heatmap showing the expression of conventional immune signatures and immune checkpoints. (**C**) Comparing the enrichment scores of immune subpopulations and immune-related signaling pathways between TLS-high and -low groups. (**D**) The correlation between TLS and the enrichment scores of each step in the cancer immunity cycle. Wilcoxon rank-sum test was performed to measure the difference between two groups.

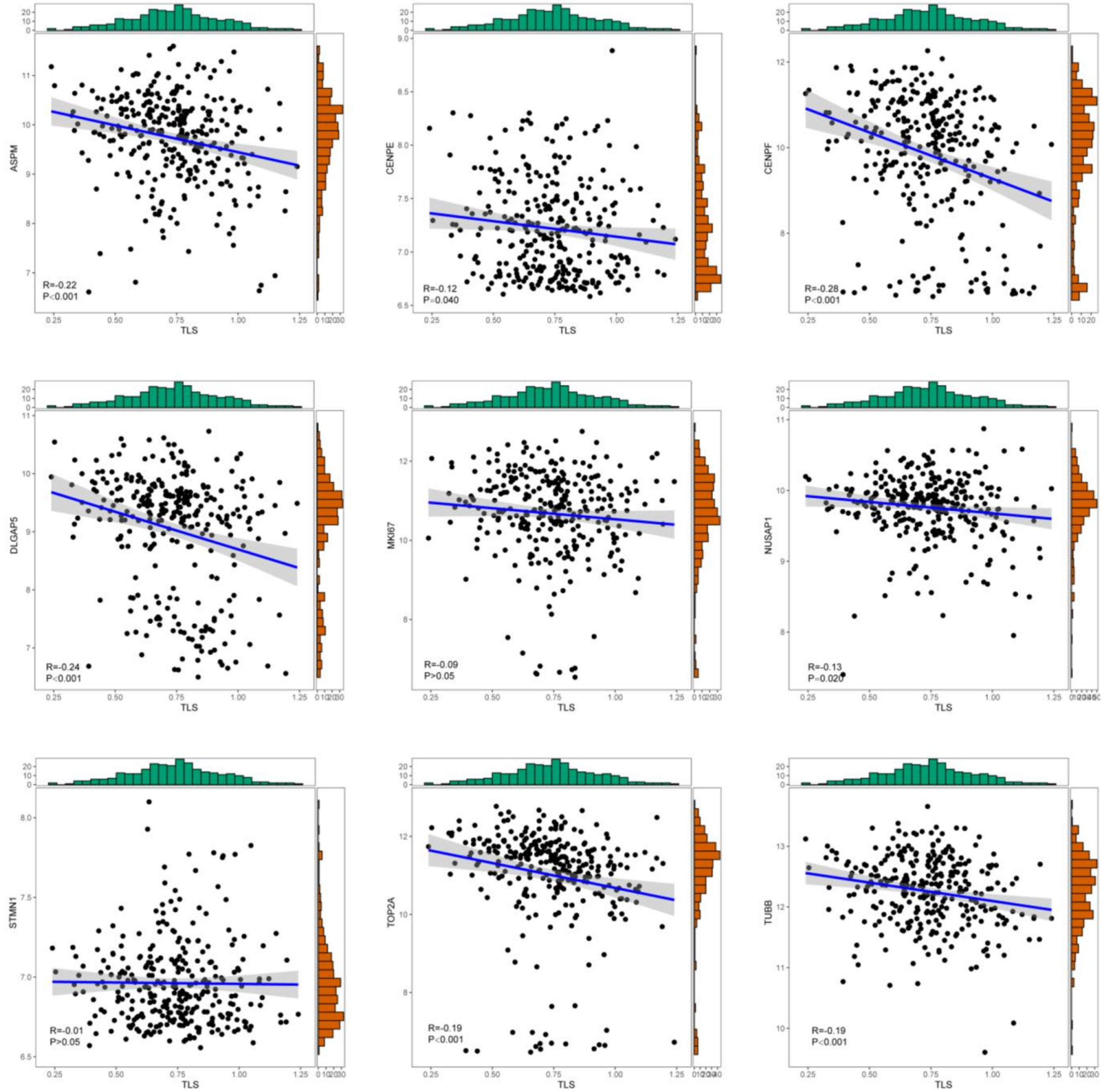

Supplementary Figure 8. Correlations between TLS score and a total of nine proliferation biomarkers in the GSE44001 cohort.

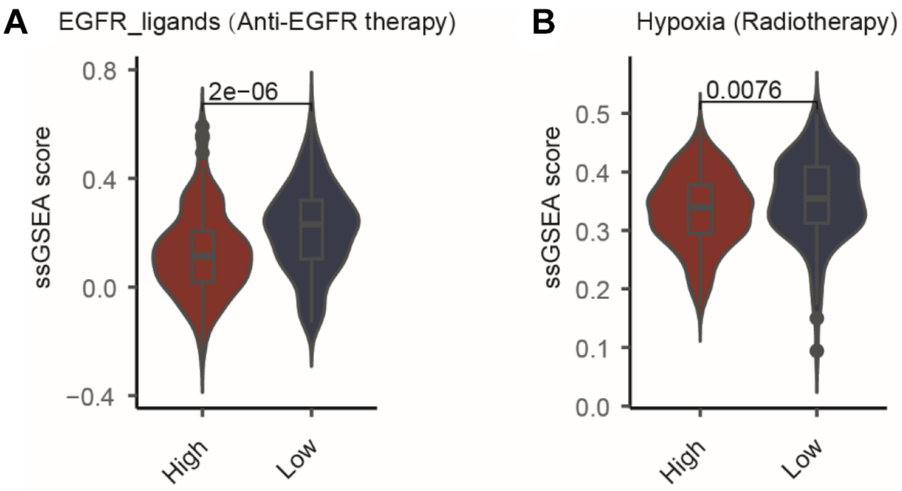

Supplementary Figure 9. Comparing the (**A**) anti-EGFR therapy and (**B**) radiotherapy-related signaling pathways between TLS-high and -low groups.
