# Supplementary material for: Tertiary lymphoid structures associated with enhanced anti-tumor immunity and favorable prognosis in cervical squamous carcinoma
Source: Aging (Albany NY). 2024 Apr 17;16(8):6898–920. doi: 10.18632/aging.205733 (PMC11087108; doi:10.18632/aging.205733)
Supplement: Supplementary Table 1 [file aging-16-205733-s002.pdf]

SUPPLEMENTARY TABLE

Supplementary Table 1. The information of published datasets used in the study.

Dataset	Cancer type	Number of samples	Platform
TCGA-CESC	CESC	292	RNA-seq
GSE44001	CESC	300	RNA-seq
GSE194040	BRCA	69	RNA-seq
PRJEB23709	Melanoma	73	RNA-seq
GSE171894	CESC	4	scRNA-seq
GSE168652	CESC	1	scRNA-seq
